# Adolescent determinants of life-course leisure-time vigorous physical activity trajectories: a 27-Year longitudinal study

**DOI:** 10.1186/s12889-023-16191-9

**Published:** 2023-06-28

**Authors:** Frida Kathrine Sofie Mathisen, Sara Madeleine Kristensen, Coral Falco, Bente Wold

**Affiliations:** 1 Department of Health Promotion and Development, University of Bergen, Bergen, Norway; 2 Department of Sport, Food and Natural Sciences, Western Norway University of Applied Sciences, Bergen, Norway

**Keywords:** Physical activity, Adolescence, Adulthood, Cohort study, Latent class growth analysis, Trajectory, Determinant

## Abstract

**Background:**

Adolescence is regarded as formative years for building the foundations for life-long health and well-being, and adolescent determinants of physical activity (PA) development is particularly interesting. Novel approaches for the study of PA development, such as group-based trajectory modelling, opens for the possibility of identifying different patterns in the relationship among several known determinants of PA. This study aimed to explore how demographic, psychological and social factors in early adolescence determine membership in four distinct leisure-time vigorous physical activity (LVPA) trajectories from 13 to 40 years.

**Methods:**

This study is based on data from the Norwegian Longitudinal Health Behaviour Study, following a cohort born in 1977 from Western Norway. Four trajectories identified using latent class growth analysis, based on self-reposted LVPA (n = 1103, 45.5% women) measured ten times from age 13 to age 40 and 17 different adolescent determinants, were used in a multivariate multinomial logistic regression.

**Results:**

We found that gender (male), VPA intentions the next year and athletic identity associated with belonging to the two trajectories reporting the highest levels of LVPA in adolescence, while VPA intentions in ten years were associated with belonging to the active trajectory compared to the decreasingly active and low active trajectories Enjoyment increased the odds of belonging to the increasingly and decreasingly active trajectories compared to the low active trajectory. In addition, two of the social determinants, mother’s PA and emotional support from father, were associated with belonging to the increasingly active trajectory when compared to the low active trajectory. Higher family income increased the odds of belonging to the increasingly active compared to the decreasingly active trajectory.

**Conclusions:**

Both demographic, psychological, and social factors were identified as determinants of LVPA trajectory membership, and the findings support previous research related to the importance of intentions, but also indicate that enjoyment, role modelling and emotional support in PA can be of great importance to LVPA promotion among adolescents.

**Supplementary Information:**

The online version contains supplementary material available at 10.1186/s12889-023-16191-9.

## Background

The positive outcomes of physical activity (PA) on mental and physical health are well recognised [1, 2]. However, globally, more than a quarter of all adults are not sufficiently physically active [3], calling for knowledge-based strategies to increase PA. Traditionally, PA has been defined as “any bodily movement produced by skeletal muscles that result in energy expenditure”(4, p. 12^6^). This definition sets PA as a mechanical act. New perspectives on PA, acknowledging the complex nature of PA and the need for ecological perspectives, call for a new definition where PA is defined as involving “people moving, acting and performing within cultural specific spaces and contexts, and influenced by a unique array of interests, emotions, ideas, instructions and relationships” (5, p. 5). This definition emphasises the importance of the context and different modes of PA. Leisure-time is the mode, or domain, of PA contributing most to the total PA in high-income countries [6]. Further, PA can be defined in relation to frequency, duration, type, and intensity. For example, activity with an intensity that makes one sweat or get out of breath is often defined as vigorous physical activity (VPA). PA at this level of intensity is thought to have specific benefits related to all-cause mortality [7].

Identifying leisure-time vigorous physical activity (LVPA) trajectories at different phases in life and examining factors related to the trajectories is essential for developing well-targeted PA promotion [8]. While the number of studies identifying trajectories in heterogeneous populations has increased, few have explored determinants of trajectory membership from adolescence to adulthood. Only two studies have been identified, focusing on demographic and socioeconomic factors [9, 10]. In the present study, we aim to expand this approach by also examining psychological and social determinants of life course LVPA trajectories from adolescence to adulthood, spanning over 27 years.

According to a review by Lounassalo et al. [8], three and five LVPA trajectories were identified in two previous studies. The majority of participants in those studies were either in a trajectory of persistently moderate or a trajectory of persistently low levels of PA, with a smaller proportion of participants displaying persistently higher PA levels. One study identified two additional trajectories: an increasingly active or a decreasingly active trajectory. Mathiesen et al. [11] identified four LVPA trajectories. These trajectories were: (a) active (n = 102, 9%), characterised by a higher level of self-reported LVPA at all measurement points compared to the other three trajectories, (b) decreasingly active (n = 276, 25%), characterised by starting at a high level at age 13, then decreasing the level of activity in adolescence and having a lower level of activity in adulthood, (c) increasingly active (n = 128, 12%), persons starting at a low level of LVPA and then increasing their activity level in adolescence and ending up at the same level as the active trajectory at age 40, and lastly, (d) low active (n = 597, 54%), consisting of more than half of the participants, characterised by a low level of LVPA in early adolescence and a consistently low level until age 40 years. In addition to identifying the four trajectories, their relation to four activity domains (membership in sports clubs, diversity in leisure-time physical activities, peer PA and outdoor recreation) were explored. Persons in the persistently active trajectory were more involved in all activity domains compared to those in the trajectory of low activity from adolescence to adulthood. Changes in sports club membership, PA among peers, outdoor recreation, and diversity in leisure-time physical activities were related to both an increase or a decrease in the level of LVPA from ages 13 to 40 [11]. The present article is a follow up of these findings, applying data from the same longitudinal study to examine the determinants of the four trajectories.

Previous research on the correlates and determinants of PA calls for a multidimensional approach to understanding PA development. While correlates point to factors that are related to the behaviour in question, determinants indicate some form of causal relationship and are best explored using longitudinal designs [12]. However hard to confirm, some level of causal determination in the relationship between the included factors and LVPA trajectory membership is assumed. Adolescent determinants related to PA development are particularly interesting as adolescence is considered formative years, as individuals gain physical, cognitive, emotional, social, and economic resources to build the foundation for later life health and well-being [13].

Several theories and models include psychological and social determinants of PA. Most prominent are the theory of planned behaviour (TPB) [14], social cognitive theory (SCT) [15] and self-determination theory (SDT) [16]. Cortis et al. [17] recommend integrating these theories to more fully understand the mechanisms underlying PA behaviour. To do so, the differences in the predictive value of the determinants of PA seem to be most informative. The intention to perform PA, as suggested by TPB, has been identified as a major cognitive determinant [17]. In line with SDT, enjoyment is the most important emotional determinant, while intrinsic motivation, identified regulation, and competence are associated with higher PA in all ages [17]. Among the more domain-specific determinants derived from SCT, self-efficacy shows a convincing positive association with overall PA. Athletic identity may also constitute an important psychological determinant of lifelong LVPA because identity plays a significant role in regulating behaviour, as outlined by SDT [18]. Seeing oneself as a PA-oriented person is related to a greater capacity for self-regulation of PA. Continued PA behaviour can lead to the incorporation of PA as a defining element of participants’ self-identity, which then serves as a motivator for continuing to be active [18]. Several studies suggest that those who favour an athletic component of self-concept are more likely to be physically active than those who do not [19].

Different conditions in the immediate social environment affect intentions to engage in PA, enjoyment and value of PA, and perceived competence in PA. Parents serve as primary agents responsible for initiating their children’s participation in PA and sports and maintain a vital role in supporting those sports experiences [2, 20]. Social support works in four different ways; evaluative, informative, emotional and instrumental [21]. Parental support in leisure activities such as youth sports and other physical activities may imply that the participant is evaluated and receives feedback on what he/she does, as well as information and knowledge about why such activity is important and how it can best be carried out. Emotional support includes, among other things, encouragement to engage in PA. Instrumental support is provided by the participant getting help to do things, for example, by parents standing up in connection with training and matches. Such support contributes to meeting young people’s need for competence and belongingness and strengthens intentions, perceived competence, and enjoyment in PA, which contributes to PA adherence among adolescents [2]. Parents may also affect their children’s PA involvement through their PA behaviour, as suggested by the principle of observational learning in SCT. Several studies show that adolescent PA is linked to parental PA, which suggests that parents are effective role models [2, 20].

By utilising more advanced analytic approaches, such as group-based trajectory modelling, one can detect latent classes and reveal different patterns of relationships among several known determinants of PA. The research literature in this area is very limited, but demographic, socioeconomic or behavioural determinants were investigated in two studies [9, 10]. Both studies found that being male increased the odds of belonging to a persistently active trajectory compared to an inactive or low active trajectory. Higher grade point averages [10], higher level of education and higher household income [9] were associated with being in the more active trajectories. Rovio et al. [10] found that parents’ PA at age 12 increased the odds of belonging to a decreasingly active trajectory compared to a persistently low active trajectory.

Gender, socioeconomic status and body mass index (BMI) have been found to relate to PA trajectory membership [8] and should therefore be included as possible demographic determinants for trajectory membership.

Consequently, this study aims to examine how demographic (gender, family income, BMI), psychological (intentions, enjoyment, self-efficacy, self-determination, competence, importance of competence, athletic identity) and social (parental physical activity, support, and encouragement) factors in early adolescence can predict membership in four distinct LVPA trajectories from 13 to 40 years.

## Methods

### Study population

The Norwegian Longitudinal Health Behaviour Study is two-generational, surveying the same group of respondents ten times (1990–2017) from age 13 to 40, and in addition, their parents three times (in 1990, 1993 and 1996). The sample was drawn from a county on the west coast of Norway and comprised students from 22 randomly selected schools (54 classes) systematically picked from an alphabetic list of schools in the region. Of the initial sample of 1195 students, 924 (78%) participated in 1990, comprising 510 boys (55%), with an average age of 13.3 years. As expected, the number of respondents decreased over the 27 years of follow-up; however, it was acceptable also at the last follow-up in 2017 with 455 respondents.

Before the first measurement point in 1990, all levels of the formal school system were contacted by mail and informed about the study, whereby they accepted the study’s goals and agreed to participate. Informed consent from parents or caregivers and approval from the local ethics committee and the Norwegian Data Inspectorate were obtained. The respondents filled in a questionnaire given during school hours at the first three measurement points, after which it was sent to the respondents through the mail. For the data collections in 2007 and 2017, the respondents were also able to fill out the survey online.

The present analysis was based on data from 1103 respondents who participated and reported on the primary outcome variable LVPA at least once over the ten measurement points. Among these 1103 respondents, 17% completed all 10 repeated measurements of LVPA; 15% nine, 11% eight, 9% seven, 10% six, 9% five, 11% four, 10% three, 5% two, and 4% completed only one measurement. Dropout analysis showed no significant differences on baseline values of LVPA or activity domains between those who dropped out of the study before age 40 and the 455 respondents who did not [11].

### Leisure-time vigorous physical activity

This was measured using a previously used item from the Health Behaviour in School-aged Children (HBSC) Study: WHO Collaborative Cross-national Study [22], which was included in the questionnaire at all ten measurement points. The question is related to PA outside school/work hours, with vigorous intensity, and maps the frequency of such activity. The question reads, «Outside school hours, how often do you do sports or exercise to the extent that you become out of breath or sweaty?» with the following response categories (coding in parenthesis): Every day (7), 4–6 times a week (5), 2–3 times a week (2.5), Once a week (1), Once a month (0.25), Less than once a month (0), and Never (0).

There are challenges related to using a single item for mapping a complex behaviour such as LVPA; however, the measure has been found to have acceptable to good reliability in an Australian sample [23] and overall good reliability in an adolescent youth sample in Norway [24]. In terms of validity, the single-item self-reported measure has been found to correlate fairly well with maximal oxygen uptake [24].

### Adolescent determinants of LVPA trajectory membership

A total of 17 different adolescent determinants were included in the analysis. All included determinants were collected at baseline in 1990 (age 13), except the information on family income, which was assessed in the questionnaire sent to parents in 1996. The wording of each question, response categories, and coding can be found in an additional file [see Additional file 1].

Three demographic determinants were included, namely gender (binary), BMI (calculated based on self-reported height and weight), and parental reported family income in 1995 (gross household income in intervals of 100 000 NOK).

Five single-item questions related to psychological determinants were used, namely intentions, perceived competence, perceived importance of competence, athletic identity, enjoyment, self-efficacy and self-determination.

Intention for future VPA was measured using two single-item questions related to intentions for performing VPA at different levels in one year or ten years respectively. Perceived competence in PA was measured as the respondents’ assessment of their skills in relation to peers, and the perceived importance of competence item was related to how much it mattered to them if they were good or bad in PA. Athletic identity was measured by asking if the respondent thought of themselves as an athlete. Enjoyment was related to affective judgement, while self-efficacy was measured concerning one’s perceived ability to execute regular PA (task-specific efficacy). Self-determination was measured by asking the respondent to which degree they could decide how much they participated in sports.

Social determinants related to parents were included, specifically, the respondents’ perception of the mother’s and father’s level of sport or exercise, whether the respondents engaged in sports and exercise with their parents, and instrumental or emotional support from parents.

### Analytical Plan

**Fig. 1:**
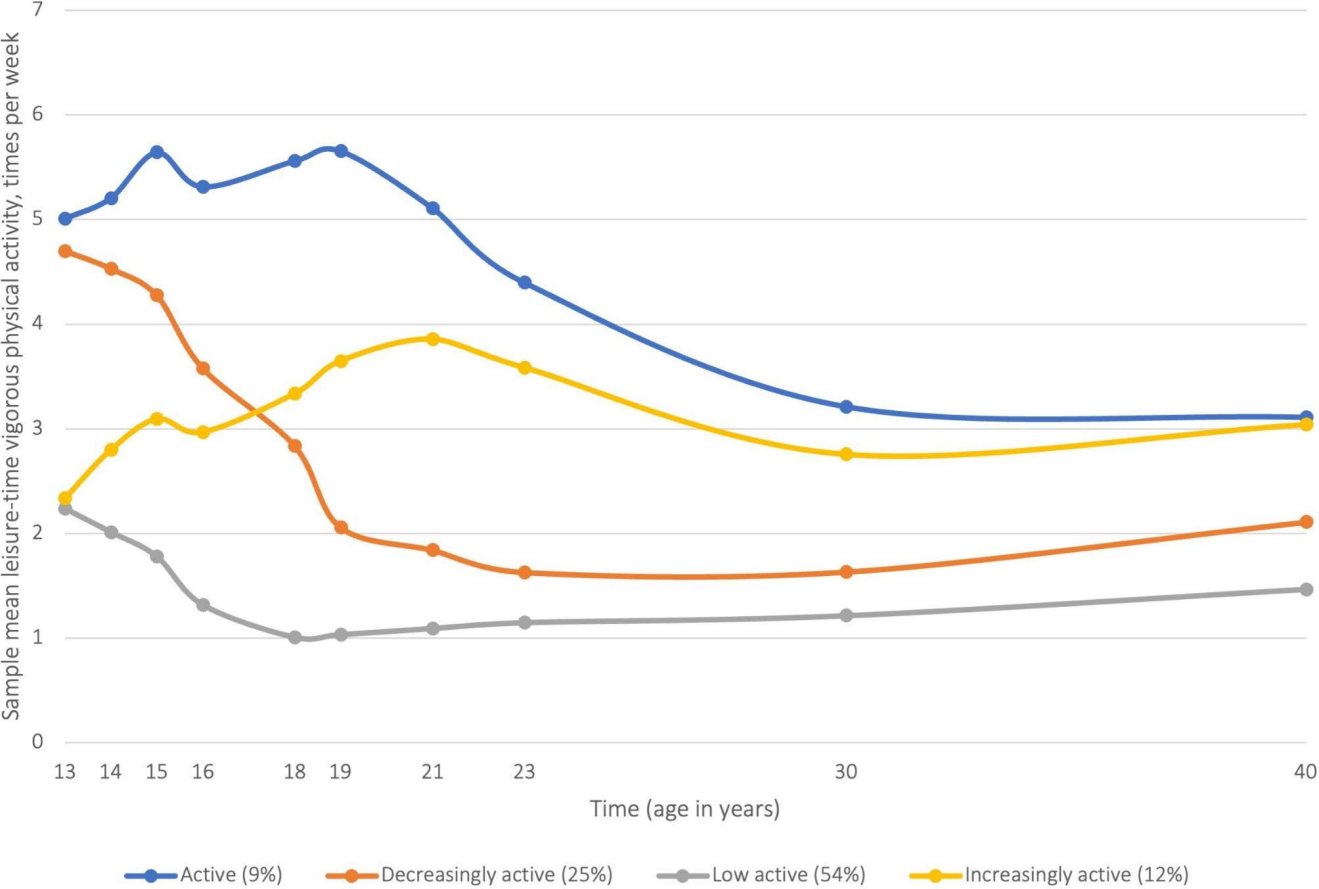
Leisure-time vigorous physical activity trajectories (n = 1103) from a latent class growth analysis showing sample mean of leisure-time vigorous physical activity (times per week) from age 13 to age 40. Reproduced from Mathisen et al. [11]. The figure is part of an open access article distributed under the terms of the Creative Commons CC BY license, and copyright permissions was not required

Latent class growth analysis (LCGA) was used to identify LVPA trajectories. The trajectory classes used in the current study have previously been identified and are presented in Fig. 1. Details about the statistical modelling can be found in an additional file [see Additional file 2] and the previously published paper [11]. We performed several multivariate multinomial regression models to investigate which characteristics were significantly predictive of trajectory group membership. First, using the ‘savedata’ command in Mplus, we created a new variable based on the likelihood of belonging to the four different latent classes. Second, demographic, psychological, and social factors were included as determinants of the latent class belonging variable. Lastly, we repeated the multivariate multinomial regression until each latent class had served as a reference group. All latent class growth curve analyses and multinomial regression models were carried out in M*plus* version 8.0 [25], using a number of auxiliary variables collected several times during the 27-year follow-up in the LCGA [11], assuming the data to be missing at random (MAR) and addressed using full information maximum likelihood (FIML) estimation to handle potential construct-level missingness [26]. The model parameters were estimated using the maximum likelihood estimator with robust standard errors (MLR).

## Results

### Descriptive statistics

The demographic, psychological, and social characteristics of the four physical trajectory groups at age 13 are presented in Table 1. The mean level and percentages followed a systematic pattern across the trajectory groups. A higher level in all variables was observed in the consistently active trajectory group, followed by lower overall means in decreasingly active, increasingly active, and low active groups consecutively.

**Table 1:** Leisure-time Vigorous Physical Activity Trajectory Distributions, Posterior-Probability Means, and Descriptive Statistics of the Study Sample

Trajectory group	Active			Decreasingly active			Increasingly active			Low active		
Distribution % (n)	9 (102)			25 (276)			12 (128)			54 (597)		
Posterior probability means	0.84			0.78			0.75			0.87		
	n	Mean/%	variance	n	Mean/%	variance	n	Mean/%	variance	n	Mean/%	variance
**Demographic**												
Woman	102	25%	0.19	276	34%	0.22	128	51%	0.25	597	54%	0.25
Family income (0–5)	65	3.35	1.47	156	3.35	1.65	79	3.53	1.17	314	3.19	1.55
BMI	68	18.07	7.83	200	17.98	3.23	84	18.25	3.44	395	18.41	5.44
**Psychological**												
VPA intention, in one year (0–7)	84	5.02	0.83	238	4.90	0.41	106	4.01	0.80	475	3.83	1.07
VPA Intention, in ten years (0–7)	83	4.69	1.18	233	4.25	1.08	105	3.76	1.06	466	3.52	1.54
Perceived competence (0–4)	82	2.49	0.64	236	2.33	0.48	107	1.94	0.39	473	1.94	0.58
Importance of competence (0–3)	85	2.61	0.40	236	2.43	0.51	108	2.32	0.50	471	2.16	0.61
Athletic identity	83	89%	0.10	228	80%	0.16	102	54%	0.25	466	39%	0.24
Enjoyment (0–4)	85	3.86	0.17	238	3.77	0.26	106	3.59	0.37	472	3.16	0.89
Self-efficacy (0–4)	84	3.29	0.73	236	3.14	0.84	106	2.91	0.69	478	2.73	0.87
Self-determination (0–4)	83	3.37	0.69	234	3.37	0.63	107	3.29	0.73	474	3.21	0.85
**Social**												
Mother’s PA (0–4)	84	1.99	1.56	234	1.74	1.43	106	1.79	1.35	473	1.48	1.57
Father’s PA (0–4)	83	2.04	1.99	227	1.97	1.67	100	1.63	1.51	463	1.51	1.75
PA together with parents (0–4)	85	1.29	1.29	236	1.06	1.06	106	0.80	0.82	482	0.77	0.81
Instrumental support 0–4)	82	2.44	1.30	236	2.32	1.50	106	1.76	1.36	466	1.59	1.52
Emotional support mother (0–4)	84	2.12	1.99	232	2.00	1.71	106	1.82	1.36	467	1.58	1.53
Emotional support father (0–4)	82	2.11	2.20	228	2.03	1.69	105	1.80	1.53	462	1.42	1.54

BMI = body mass index, PA = physical activity, VPA = vigorous physical activity.Range is presented in parenthesis. The variable categories are found in Additional file 1

### Adolescent determinants of trajectory class membership

The odds ratio results of the multivariate multinomial regression models are presented in Table 2.

**Table 2:** Adolescent determinants of leisure-time vigorous physical activity trajectories

	Decreasingly active vs active^a^			Increasingly active vs low active^b^			Decreasingly active vs increasingly active^c^			Active vs increasingly active^c^			Active vs low active^b^			Decreasingly active vs low active^b^		
	OR	95% CI		OR	95% CI		OR	95% CI		OR	95% CI		OR	95% CI		OR	95% CI	
**Demographic**																		
Woman	1.35	0.78,	2.36	0.85	0.56,	1.30	**0.57**	**0.34**,	**0.94**	**0.42**	**0.22**,	**0.81**	**0.36**	**0.20**,	**0.63**	**0.48**	**0.33**,	**0.71**
Family income	0.98	0.75,	1.28	1.23	0.99,	1.52	**0.78**	**0.62**,	**0.98**	0.80	0.58,	1.09	0.98	0.73,	1.31	0.96	0.79,	1.16
BMI	0.91	0.78,	1.06	1.00	0.90,	1.11	0.95	0.84,	1.07	1.04	0.88,	1.23	1.04	0.89,	1.21	0.95	0.86,	1.04
**Psychological**																		
VPA intention, in one year	1.19	0.67,	2.10	0.82	0.62,	1.10	**4.67**	**3.08**,	**7.07**	**3.94**	**1.92**,	**8.09**	**3.24**	**1.68**,	**6.25**	**3.84**	**2.78**,	**5.31**
VPA Intention, in ten years	**0.65**	**0.47**,	**0.90**	1.12	0.89,	1.40	0.94	0.72,	1.24	1.45	0.99,	2.12	**1.62**	**1.15**,	**2.28**	1.05	0.85,	1.30
Perceived competence	0.83	0.56,	1.22	**0.65**	**0.44**,	**0.97**	1.34	0.87,	2.07	1.62	0.97,	2.71	1.05	0.69,	1.61	0.87	0.64,	1.20
Importance of competence	0.81	0.52,	1.27	1.00	0.73,	1.37	0.70	0.48,	1.02	0.86	0.52,	1.42	0.86	0.56,	1.34	**0.70**	**0.52**,	**0.93**
Athletic identity	0.63	0.26,	1.53	1.11	0.63,	1.97	**2.30**	**1.16**,	**4.58**	**3.68**	**1.37**,	**9.92**	**4.09**	**1.70**,	**9.83**	**2.55**	**1.54**,	**4.23**
Enjoyment	0.79	0.38,	1.64	**2.52**	**1.72**,	**3.68**	0.63	0.39,	1.04	0.80	0.37,	1.74	2.01	0.99,	4.08	**1.59**	**1.09**,	**2.33**
Self-efficacy	0.94	0.68,	1.31	1.01	0.77,	1.32	0.95	0.68,	1.31	1.01	0.67,	1.51	1.02	0.72,	1.43	0.95	0.76,	1.20
Self-determination	1.08	0.76,	1.53	1.07	0.84,	1.36	1.05	0.78,	1.41	0.97	0.65,	1.45	1.04	0.72,	1.49	1.12	0.89,	1.41
**Social**																		
Mother’s PA	0.83	0.65,	1.06	**1.24**	**1.01**,	**1.53**	0.82	0.65,	1.05	1.00	0.74,	1.34	1.24	0.96,	1.59	1.02	0.86,	1.22
Father’s PA	1.20	0.93,	1.54	0.95	0.78,	1.16	1.24	0.98,	1.56	1.04	0.76,	1.41	0.98	0.75,	1.29	1.17	0.98,	1.41
PA together with parents	0.83	0.61,	1.12	0.89	0.67,	1.18	1.05	0.77,	1.44	1.27	0.87,	1.85	1.12	0.82,	1.55	0.93	0.74,	1.18
Instrumental support	1.07	0.81,	1.41	0.91	0.74,	1.11	1.26	0.99,	1.60	1.18	0.87,	1.61	1.07	0.82,	1.40	1.15	0.95,	1.38
Emotional support mother	0.97	0.69,	1.36	0.97	0.76,	1.25	1.07	0.81,	1.42	1.11	0.75,	1.62	1.08	0.77,	1.50	1.05	0.86,	1.27
Emotional support father	1.11	0.79,	1.55	**1.29**	**1.00**,	**1.67**	0.77	0.58,	1.03	0.70	0.47,	1.03	0.90	0.65,	1.26	1.00	0.81,	1.23

BMI = body mass index, PA = physical activity, VPA = vigorous physical activity

The values are odds ratios (OR) and 95% confidence intervals (95% CI). All determinants are entered simultaneously. Statistically significant (P < .05) results are marked with bold font

^a^Active trajectory was used as the reference category

^b^Low active trajectory was used as the reference category

^c^Increasingly active trajectory was used as the reference category

### Demographic factors

Women were found to have decreased odds of being active compared to increasingly active (OR = 0.42, 95% CI = 0.22, 0.81) and low active (OR = 0.36, 95% CI = 0.20, 0.63). Additionally, women had lower odds of being decreasingly active than increasingly active (OR = 0.57, 95% CI = 0.34, 0.94) or low active (OR = 0.48, 95% CI = 0.33, 0.71).

Higher parental reported family income lowered the odds of being decreasingly active compared to increasingly active (OR = 0.78, 95% CI = 0.62, 0.98).

### Psychological factors

Intentions of higher levels of VPA in one year at age 13 increased the odds of being active rather than increasingly active (OR = 3.94, 95% CI = 1.92, 8.09) or low active (OR = 3.24, 95% CI = 1.68, 6.25). Those with intentions of higher levels of VPA in adolescence also had higher odds of being decreasingly active than increasingly active (OR = 4.67, 95% CI = 3.08, 7.07) or low active (OR = 3.84, 95% CI = 2.78, 5.31).

Youth with higher intentions of VPA in ten years had lower odds of being decreasingly active than active (OR = 0.65, 95% CI = 0.47, 0.90) and also had higher odds of being active compared to low active (OR = 1.62 95% CI = 1.15, 2.28).

People with higher perceived competence were less likely to be increasingly active than low active (OR = 0.65, 95% CI = 0.44, 0.97). Participants with higher perceived importance of competence had lower odds of belonging to the decreasingly active compared to the low active trajectory (OR = 0.70, 95% CI = 0.52, 0.93).

People who identified as an athlete had higher odds of being active than increasingly active (OR = 3.68, 95% CI = 1.37, 9.92) or low active (OR = 4.09, 95% CI = 1.70, 9.83). Additionally, they had higher odds of being decreasingly active than low active (OR = 2.55, 95% CI = 1.54, 4.23) and increasingly active (OR = 2.30, 95% CI = 1.16, 4.58).

Participants who enjoyed sports had higher odds of being increasingly active than low active (OR = 2.52, 95% CI = 1.72, 3.68). They were also more likely to be decreasingly active than low active (OR = 1.59, 95% CI = 1.09, 2.33).

### Social factors

People with a physically active mother had higher odds of being in the increasingly active trajectory class than the low active trajectory class (OR = 1.24, 95% CI = 1.01, 1.53). Participants who experienced emotional support from their father had higher odds of being increasingly active than low active (OR = 1.29, 95% CI = 1.00, 1.67).

## Discussion

In this Norwegian cohort, we identified adolescent determinants for membership in four distinct LVPA trajectories from adolescence to adulthood. Intentions for future VPA in young adulthood were associated with belonging to the active trajectory compared to the decreasingly active or low active trajectories, while higher levels of enjoyment increased the odds of belonging to the increasingly or decreasingly active trajectories compared to the low active trajectory. In addition, two of the social determinants, mother’s PA and emotional support from father, were associated with belonging to the increasingly active trajectory when compared to the low active trajectory.Lastly, women were less likely to follow the active and decreasingly active trajectories.

Those in the active and decreasingly active trajectories reported similar mean levels on all the determinants. The increasingly active and low activity trajectories also reported similar levels, but consistently lower than those in the active or decreasingly active trajectory groups. This finding may reflect their position at the start of each trajectory, as the LVPA level at age 13 was higher (about 5 times per week) among those in the active and decreasingly active trajectories than in the other two trajectories (about 2.3 times per week) [11]. Thus, they had more familiarity with LVPA and therefore had more opportunities for experiencing enjoyment, self-efficacy, self-determination, competence, perceived importance of competence, athletic identity, and developing intentions, explaining the higher mean levels on almost all the included determinants.

The main distinction between the active versus the decreasingly active trajectories seems to be that those in the former trajectory were more likely to report a higher intention of being physically active in the future, i.e. in young adulthood. This finding is corroborated by the conclusions in a review of determinants of PA [17] and supports the TPB [14]. Those in the active trajectory seem to have experienced a sufficient degree of actual control over engagement in PA over the years, as they have been able to carry out their PA intentions over 27 years.

Among the two trajectory groups starting at the lower end of the scale, those belonging to the increasingly active trajectory reported slightly higher mean levels on all the included determinants than those in the low active trajectory. Higher levels of enjoyment increased the odds of belonging to the increasingly active trajectory. In line with SDT, enjoyment increases the motivation to perform PA because it is intrinsically rewarding.

Enjoyment can be affected by the immediate social environment, where parents are important role models and primary agents for contributing to children’s participation in PA and sports. In addition to the increased odds related to enjoyment, we also found that higher levels of mother’s PA and emotional support from father increased the odds for belonging to the increasingly active trajectory compared to the low active trajectory.

Exposure to active role models can make the individual more aware of opportunities to engage in LVPA. Increased attention can lead to more positive attitudes towards the activity, which in turn can affect the individual’s motivation to initiate and maintain LVPA. Multiple studies on the connection between parental and adolescent PA support that this might be important for PA development [2, 20]. The influence of parental PA on LVPA trajectory membership can, in addition to modelling, also be related to support and encouragement as suggested by previous studies [27] [28].

The results showed a small decrease in the odds of belonging to the increasingly active vs. low active trajectory in relation to higher perceived competence. Due to the documented effect of perceived competence on PA behaviour [17, 29], this unexpected finding may be interpreted carefully due to the small difference in means and large differences in group size between the two trajectory groups. However, the measure of perceived competence was directed at comparing their competence to others, as opposed to other determinants, such as enjoyment, which was internally directed. This might have contributed to the results, as the intrinsic motivation determinants were contributing more to positive development in LVPA. Similarly, we found that higher perceived importance of competence in PA associated with belonging to the low active trajectory compared to the decreasingly active trajectory. We hypothesise that this might also be related to the measure, as the question is related to which degree you care whether you are eighter good or bad in sports. It is also possible that this item is not as related to the level of LVPA as other factors such as intentions or enjoyment, because the perceived importance of competence in sports can be related to other more external factors, such as social comparison or cultural importance, rather than the individual’s competence, motivation for or engagement in sports.

There was a gender difference among the trajectories, and being a woman reduced the odds of belonging to the active and the decreasingly active trajectories. Thus, there were more men in the active and decreasingly active LVPA trajectories, which is consistent with earlier reports [10, 30], and studies showing a larger decrease in PA among boys than girls during adolescence [31].

Lastly, we found that higher levels of parental reported family income gave a slight increase in the odds of belonging to the increasingly active compared to the decreasingly active trajectory. Higher family income may be related to having more time and resources to support and encourage those in the increasingly active trajectory to engage and maintain their LVPA involvement throughout the adolescent years.

### Limitations

One limitation of this study might be the single-item measurements of the adolescent determinants and frequency of LVPA. Using a single item to measure LVPA may have oversimplified a phenomenon with many dimensions, such as type of activity or duration. Single indicators have decreased levels of detail, unknown reliability, and might not capture complex psychological constructs [32]. Thus, because self-efficacy and self-determination are commonly measured as latent constructs, we advise caution in interpreting the results pertaining to the predictive power of these factors. However, the other determinants measured in this study are not assumed to be complex constructs but rather one-dimensional, clear, and simple questions pertaining to personal enjoyment, intentions, and beliefs of sports participation, as well as the availability of environmental resources. As Bowling [33] notes: if one question works, why ask several? If single items appropriately measure what they are meant to assess, multiple indicator scales might not necessarily be favourable over single indicators [32]. We argue that the indicators in this study have strong face validity. Indeed, items are worded similarly to indicators in scales, such as the Athletic identity measurement scale (AIMS) [34] and the Physical Activity Enjoyment Scale (PACES) [35]. Furthermore, single-item measures are vastly more cost-effective and parsimonious regarding efficiency and utility [32], which are crucial aspects in longitudinal studies spanning 27 years or more.

The usage of self-reported data in this study might be considered another limitation. For one, people might under or overreport due to social desirability [36, 37], which can lead to a misrepresentation of the data. However, the indicators in this study were designed to be as neutral as possible [38], except for the perceived competence indicator, wherein participants are to compare themselves to peers regarding sports proficiency. The survey and administration were designed to be non-threatening and self-administered [38]. Participants were given random numbers for their identification instead of their names and put the completed surveys in sealed envelopes. Secondly, common method bias (e.g., [39]) might pose a threat to the discriminative validity of the measurements in the data. For instance, it is possible that the variance in the adolescent determinants measured in this study is explained by one single latent construct. To examine this possibility, we performed a post hoc Harman’s single-factor test [40], which included all determinants in the study. The results clearly showed that several latent factors were extracted, indicating that common method variance does not pose a large threat to the discriminative validity of the variables in this study.

Another limitation of the study might be that the sample is not representative of the Norwegian population. However, because the study is of such magnitude in terms of duration and resource expenditure, it would be near impossible to follow up on a more extensive sample size over 27 years. Further, the baseline mean of LVPA collected in October/November 1990 was almost identical to the nationally representative HBSC study sample of Norwegian 13-year-olds (n = 1616) in November 1989. Hence the sample from western Norway at baseline was representative of the relevant age group in Norway [41].

Following the same group of individuals over 27 years entails challenges related to wave nonresponse. The level of missing data was high. However, dropout analyses were conducted [11] and the missing data were assumed to be MAR and handled using FIML with several auxiliary variables in the analysis, which has been shown to reduce the chance of inadvertently omitting an important cause of missingness, and also increases efficiency and reduces bias [42]. Lastly, group-based modelling reduces by approximation to summarise data, comparing individuals that are not entirely homogenous. It is important to recognise that the analysis gives only the probability of following a trajectory.

## Conclusions

Demographic, psychological, and social determinants for membership in LVPA trajectories were identified in this longitudinal study with a 27-year follow-up. Men, those with a stronger athletic identity and with intentions of higher levels of VPA the next year were more likely to belong to the two trajectories starting with the highest levels of LVPA at age 13. Comparing these two trajectories, higher intentions for VPA in ten years increased the odds of belonging to the active compared to the decreasingly active trajectory. Enjoyment increased the odds of belonging to the increasingly active trajectory compared to the low active. Supporting the development of intentions for and the experience of enjoyment in PA during adolescence may promote persistence in or increase such health-promoting behaviour during the crucial and formative years of behavioural development.

We argue that the findings provide valuable information on how determinants at a young age might impact physical activity behaviour in a life course perspective. For example, the implications of the findings in this study might be relevant to the field of PA and health promotion practitioners aiming to increase physical activity across the lifespan. However, as our results are related to leisure-time and VPA only, future research should also study the relationship between trajectories for moderate or total PA and adolescent determinants.

## Electronic supplementary material

Below is the link to the electronic supplementary material.

Additional file 1: Questionnaire items

Additional file 2: Latent class growth analysis

## Data Availability

The data and materials used for the current study are available from the corresponding author upon reasonable request.

## List of Abbreviations

BMI: Body mass index
FIML: Full information maximum likelihood
HBSC: The Health Behaviour in School-aged Children (HBSC) Study:WHO Collaborative Cross-national Study
LCGA: Latent class growth analysis
LVPA: Leisure-time vigorous physical activity
MAR: Missing at random
PA: Physical activity
VPA: Vigorous physical activity
