# Supplementary material for: Adolescent determinants of life-course leisure-time vigorous physical activity trajectories: a 27-Year longitudinal study
Source: BMC Public Health. 2023 Jun 28;23:1258. doi: 10.1186/s12889-023-16191-9 (PMC10308619; doi:10.1186/s12889-023-16191-9)
Supplement: Supplementary file 1 — Additional file 1: Questionnaire items [file 12889_2023_16191_MOESM1_ESM.pdf]

Additional file 1

Questionnaire items
A list of all questionnaire items used to measure the included determinants, providing the wording all items, response categories and coding used in the analysis.

Questionnaire Item	Response categories	Coding
Demographic		
Are you a boy or a girl?	Boy	0
	Girl	1
Height	cm	
Weight	kg	
What was your household's total income in 1995 (gross)?	Less than 100.000	0
	100-199.000 or more	1
	200-299.000	2
	300-399.000	3
	400-499.000	4
	500.000 or more	5
Psychological		
Think about yourself in one year. How many times per week do you think you will do sports or exercise to the extent that you become out of breath or sweat?	Every day	7
	4-6 times a week	5
	2-3 times a week	2,5
	Once a week	1
	Once a month	0,25
	Less than once a month	0
	Never	0
Think about yourself in ten years. How many times per week do you think you will do sports or exercise to the extent that you become out of breath or sweat?	Every day	7
	4-6 times a week	5
	2-3 times a week	2,5
	Once a week	1
	Once a month	0,25
	Less than once a month	0
	Never	0
Think about many different sports outside school. How good are you normally in sports?	Better than peers	4
	Slightly better than peers	3
	As peers	2
	Slightly worse than peers	1
	Worse than peers	0
How much does it matter to you whether you are good or bad at sports?	It means a lot	3
	It means some	2
	It means little	1
	It doesn't mean anything	0
Do you think of yourself as a sports girl/boy?	Yes	1
	No	0

	How do you enjoy doing sports?	Very much	4
		Enjoy it	3
		Neither good nor bad	2
		Bad	1
		Very bad	0
	How easy would it be for you to engage in sports or exercise, should you wish to do so?	Very easy	4
		Easy	3
		Neither easy nor difficult	2
		Difficult	1
		Very difficult	0
	Do you feel that you can decide for yourself how much you are going to participate in sports?	I decide for myself a great deal	4
		I can decide for myself a lot	3
		I can decide for myself to some degree	2
		I can decide for myself a little	1
		I cannot decide for myself much	0
Social			
	Do your mother/father do sports or exercise?	4 times per week or more	4
		2-3 times a week	3
		Once a week	2
		Less than once a week	1
		Never	0
		I don't have a mother/father	Missing
	Do you happen to do sports or exercise together with your mother or father?	4 times per week or more	4
		2-3 times a week	3
		Once a week	2
		Less than once a week	1
		Never	0
	Does it happen that your parents help you do sports? (It can be such things as driving or picking you up, timing, keeping scores or similar.)	4 times per week or more	4
		2-3 times a week	3
		Once a week	2
		Less than once a week	1
		Never	0
	How often do your mother/father encourage you to do sports or exercise to keep fit?	4 times per week or more	4
		2-3 times a week	3
		Once a week	2
		Less than once a week	1
		Never	0
