# Supplementary material for: Adolescent determinants of life-course leisure-time vigorous physical activity trajectories: a 27-Year longitudinal study
Source: BMC Public Health. 2023 Jun 28;23:1258. doi: 10.1186/s12889-023-16191-9 (PMC10308619; doi:10.1186/s12889-023-16191-9)
Supplement: Supplementary file 2 — Additional file 2: Latent class growth analysis [file 12889_2023_16191_MOESM2_ESM.pdf]

Additional file 2

Latent class growth analysis

Before performing the latent class growth analysis (LCGA) in Mplus, version 8.7, data on leisure-time vigorous physical activity (LVPA) collected at all ten measurement points were modelled in latent growth models to test what number of growth parameters best fitted the data. The three models ranged from two to four growth parameters, where the best fit was obtained when the model included four growth parameters (intercept, slope, quadratic, and cubic slope factor). This was determined by the highest comparative fit index (CFI=0.911) and the lowest root mean square error of approximation (RMSEA=0.05). The same data and number of growth parameters were used in the LCGA.

LCGA is a type of group-based trajectory model, making it possible to identify classes of individuals based on their joint growth over time (1). The LCGA was performed by treating the ten measures of LVPA as continuous and together with several auxiliary variables related to activity domains (e.g., membership in sports clubs, diversity in leisure-time activity, peer PA) collected in adolescence, young adulthood and adulthood.

Data were assumed to be missing at random (MAR) and handled using full information maximum likelihood estimation (FIML). The maximum likelihood estimator with robust standard errors (MLR) was used when estimating the model parameters. To determine the fit and interpretability of the model and number of latent trajectory classes the Akaike information criteria (AIC), Bayesian information criteria (BIC), entropy, average posterior probability >0.70 for within-group membership, the Vuong–Lo–Mendell–Rubin test (VLMR), and the bootstrap likelihood ratio test (BLRT) were used (2). The model fit for two, three, four, five, six and seven latent trajectory classes were assessed. The final class enumeration was based on entropy and posterior probability of class membership, along with theoretical and empirical support for the four-class solution.

References

1. Nagin DS. Group-Based Trajectory Modeling: An Overview. Ann Nutr Metab. 2014;65(2–3):205–10.

2. Schoot R van de, Sijbrandij M, Winter SD, Depaoli S, Vermunt JK. The GRoLTS-Checklist: Guidelines for Reporting on Latent Trajectory Studies. Structural Equation Modeling: A Multidisciplinary Journal. 2017 May 4;24(3):451–67.
